# Relaxant Action of *Plumula Nelumbinis* Extract on Mouse Airway Smooth Muscle

**DOI:** 10.1155/2015/523640

**Published:** 2015-02-11

**Authors:** Li Tan, Weiwei Chen, Ming-Yu Wei, Jinhua Shen, Meng-Fei Yu, Guangzhong Yang, Donglin Guo, Gangjian Qin, Guangju Ji, Qing-Hua Liu

**Affiliations:** ^1^Institute for Medical Biology & Hubei Provincial Key Laboratory for Protection and Application of Special Plant Germplasmin Wuling Area of China, College of Life Sciences, South Central University for Nationalities, Wuhan 430074, China; ^2^College of Pharmacy, South-Central University for Nationalities, Wuhan 430074, China; ^3^Lankenau Institute for Medical Research & Main Line Health Heart Center, 100 Lancaster Avenue, Wynnewood, PA 19096, USA; ^4^Department of Medicine-Cardiology, Feinberg Cardiovascular Research Institute, Northwestern University Feinberg School of Medicine, Chicago, IL 60611, USA; ^5^National Laboratory of Biomacromolecules, Institute of Biophysics, Chinese Academy of Sciences, Beijing 100101, China

## Abstract

The traditional herb *Plumula Nelumbinis* is widely used in the world because it has many biological activities, such as anti-inflammation, antioxidant, antihypertension, and butyrylcholinesterase inhibition. However, the action of *Plumula Nelumbinis* on airway smooth muscle (ASM) relaxation has not been investigated. A chloroform extract of *Plumula Nelumbinis* (CEPN) was prepared, which completely inhibited precontraction induced by high K^+^ in a concentration-dependent manner in mouse tracheal rings, but it had no effect on resting tension. CEPN also blocked voltage-dependent L-type Ca^2+^ channel- (VDCC-) mediated currents. In addition, ACh-induced precontraction was also completely blocked by CEPN and partially inhibited by nifedipine or pyrazole 3. Besides, CEPN partially reduced ACh-activated nonselective cation channel (NSCC) currents. Taken together, our data demonstrate that CEPN blocked VDCC and NSCC to inhibit Ca^2+^ influx, resulting in relaxation of precontracted ASM. This finding indicates that CEPN would be a candidate of new potent bronchodilators.

## 1. Introduction

Asthma and chronic obstructive pulmonary disease (COPD) are highly prevalent diseases that currently affect more than 300 million individuals worldwide. Excessive airway obstruction is a cardinal symptom in asthma and COPD [1–3]. Airway smooth muscle cells (ASMCs), one important cell type in the respiratory system, contribute to the symptoms of these airway obstructive diseases [4]. Excessive contraction of ASMCs can narrow the airway lumen, which limits gas exchange and threatens the lives of asthmatics and COPD patients [5, 6]. Therefore, bronchodilators, such as *β*2 adrenergic agonists, are standard medicines that are widely used for the pharmacological management of these diseases [7, 8]. However, the currently available bronchodilators have serious side effects; thus, the development of novel effective and safe bronchodilators is an important task for asthma and COPD therapy.

Some of traditional herbs have long been used in the treatment of asthma and COPD because they can effectively relax ASM contraction [9–11]. This inspired us to identify novel bronchodilators from the traditional herbs. This study investigated whether an extract of* Plumula Nelumbinis* relaxed precontracted ASM induced by high K^+^ and ACh and the underlying mechanism.

*Plumula Nelumbinis* is the green germ of a mature lotus (*Nelumbo nucifera* Gaertn) seed [12, 13]. This traditional medicine was documented in a well-known materia medica “Bencao Gangmu” by Shizhen Li during the Ming dynasty (1596).* Plumula Nelumbinis* has many pharmacological and physiological activities, including anti-inflammation [14, 15], antioxidant [16], antihypertension [17], and butyrylcholinesterase inhibition [18]. Several active ingredients in* Plumula Nelumbinis* exhibit beneficial biological activities on smooth muscle: neferine markedly inhibits angiotensin II-stimulated proliferation, reduces ^45^Ca-influx induced by phenylephrine in vascular smooth muscle [19, 20], and relaxes corpus cavernosum smooth muscle cells [21], and isoliensinine possesses antiproliferative effects on coronary arterial smooth muscle cells [22, 23]. However, the effects of* Plumula Nelumbinis* extract on ASMC tension have not been studied previously.

The present study investigated the relaxation effects of a chloroform extract of* Plumula Nelumbinis* (CEPN) on mouse ASM precontraction induced by high K^+^ and ACh. The results show that CEPN inhibited VDCCs and NSCCs, which then resulted in relaxation of precontracted ASM.

## 2. Materials and Methods

### 2.1. Plant Material

*Plumula Nelumbinis*, germs of* Nelumbo nucifera* Gaertn seeds, were collected in Honghu City, Hubei Province, China, in October 2013, and were identified by Professor Dr. Ding-rong Wan, College of Pharmacy, South-Central University for Nationalities. A voucher specimen (SCUN201310006) is deposited at the Herbarium of the College of Pharmacy, South-Central University for Nationalities, China.

### 2.2. Extraction and Isolation

*Plumula Nelumbinis* (1 Kg) were air-dried, milled into powder, and extracted at room temperature with 95% ethanol (3 × 4 L, 2 h each). Extracts were centrifuged, and the supernatants were collected. The ethanol extract (193 g) was next evaporated to dryness under reduced pressure using a rotary evaporator and immersed in a 2% HCl solution (500 mL). Residues were extracted with petroleum ether (3 × 300 mL, 4 h each) to remove lipids. Ammonia adjusted the pH of the filtrate to 10, and the crude sample was extracted with chloroform (5 × 300 mL, 4 h each). The chloroform extract was evaporated under reduced pressure, and the extraction yield was 0.71% of the raw material dry weight. The dried chloroform extract of* Plumula Nelumbinis* (CEPN) was dissolved in 3% DMSO for the experiments.

### 2.3. Reagents

Nifedipine, acetylcholine chloride (ACh), niflumic acid (NA), tetraethylammonium chloride (TEA), and pyrazole 3 (Pyr3) were purchased from Sigma Chemical Co. (St. Louis, MO, USA). Other chemicals were purchased from Sinopharm Chemical Reagent Co. (Shanghai, China).

### 2.4. Animals

Sexually mature male BALB/c mice were purchased from the Hubei Provincial Center for Disease Control and Prevention (Wuhan, China). Mice were housed at room temperature (20–25°C) and constant humidity (50–60%) under a 12 h light-dark cycle in an SPF grade laboratory. The animal study was performed according to the guidelines of the Institutional Animal Care and Use Committee of the South-Central University for Nationalities (Wuhan, China) and approved by the Animal Care and Ethics Committee of the South-Central University for Nationalities (QHL-2, 02-03-2012).

### 2.5. ASM Contraction Measurement

Mouse ASM contraction was measured in tracheal rings [24, 25]. Mice were sacrificed using an intraperitoneal injection of sodium pentobarbital (150 mg/kg), and tracheae were isolated and quickly transferred to ice cold PSS (composition in mM: NaCl 135, KCl 5, MgCl_2_ 1, CaCl_2_ 2, HEPES 10, glucose 10, pH 7.4). Connective tissues were removed, and small rings (~5 mm) were cut from the bottom of tracheae. Each ring was mounted with a preload of 0.5 g in an organ bath with a 10 mL capacity containing PSS bubbled with 95% O_2_-5% CO_2_ at 37°C. Tracheal rings were equilibrated for 60 min, precontracted with high K^+^ (80 mM) or ACh (10^−4^ M), washed, and rested 3 times. Experiments were performed following an additional 30 min rest.

### 2.6. Isolation of Single ASMCs

Mouse ASMCs were isolated as previous method [25, 26]. Briefly, tracheae were isolated as described above and digested in an ice-cold low-Ca^2+^ physiological saline solution (LCPSS) (composition in mM: NaCl 135, KCl 5, MgSO_4_ 1, glucose 10, HEPES 10, CaCl_2_ 0.1, pH 7.4) containing 1 mg/mL papain, 0.5 mg/mL dithioerythritol, and 1 mg/mL bovine serum albumin (BSA) at 37°C for 20 min. Tissues were transferred to LCPSS containing 1 mg/mL collagenase H, 1 mg/mL dithiothreitol, and 1 mg/mL BSA and incubated at 37°C for 20 min. Tissues were washed and gently triturated in LCPSS to yield single ASMCs for experiments.

### 2.7. Measurement of VDCC Currents

VDCC currents were measured using Ba^2+^ as the charge carrier and an EPC-10 patch-clamp amplifier (HEKA, Lambrecht, Germany). The pipette solution contained the following (in mM): CsCl 130, EGTA 10, MgCl_2_ 4, Mg-ATP 4, HEPES 10, TEA 10, and pH 7.2 (adjusted with CsOH). The composition of the bath solution was (in mM) NaCl 105, CsCl 6, BaCl_2_ 27.5, glucose 11, HEPES 10, TEA-Cl 10, NA 0.1, and pH 7.4 (adjusted with NaOH). ASMCs were patched and held at −70 mV. Currents were measured following depolarization for 1000 ms from −70 to +30 mV in 10 mV increments every 10 s.

### 2.8. Measurement of NSCC Currents

The pipette solution contained the following chemicals for the measurement of NSCC currents (in mM): CsCl 18, cesium acetate 108, MgCl_2_ 1.2, HEPES 10, EGTA 3, CaCl_2_ 1, and pH 7.2 (adjusted with Tris). The free Ca^2+^ concentration was approximately 70 nM, as calculated using WEBMAXC STANDARD. The bath solution was PSS without K^+^ containing 10 *µ*M nifedipine, 100 *µ*M NA, and 10 mM TEA to block VDCC, Cl^−^, and K^+^ currents, respectively. ACh-induced NSCC currents were recorded with a ramp using a perforated whole-cell configuration with a holding potential of −60 mV. The ramp was performed over 500 ms from −80 to +60 mV.

### 2.9. Statistical Analysis

Statistical analysis was performed with Student's *t*-test using Origin 9.0 software (OriginLab, Northampton, USA). Statistical significance was defined as *P* < 0.05. Data are expressed as the means ± SEM.

## 3. Results

### 3.1. CEPN Inhibits High K^+^-Induced Precontraction

**Figure 1:**
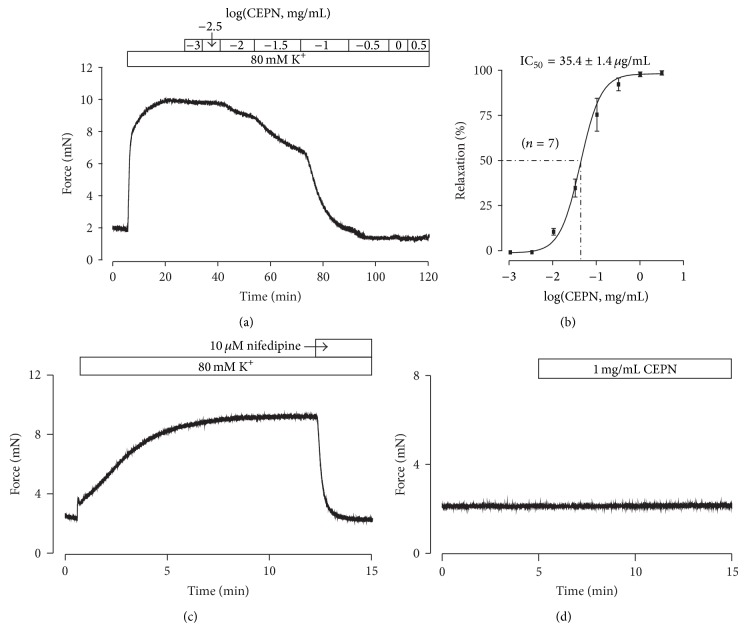
Relaxant effects of CEPN on high K^+^-induced precontraction. (a) High K^+^ induced a steady-state contraction in a mouse tracheal ring, which was inhibited by CEPN in a concentration-dependent manner. (b) Dose-relaxation curve of CEPN based on the results of 7 different experiments shown in (a). (c) High K^+^-induced precontraction was completely blocked by nifedipine. This experiment was performed in 8 tracheal rings from 8 mice, and the result was reproducible across experiments. (d) CEPN had no effect on resting tension in 4 rings. These results indicate that the CEPN-induced relaxation might result from blockade of VDCCs.

We observed the effects of CEPN on high K^+^-induced ASM precontraction to investigate whether CEPN relaxed ASM. High K^+^ (80 mM) induced contraction in a mouse tracheal ring, and CEPN (1 *µ*g–3.16 mg/mL) was cumulatively added to the organ bath. ASM contraction was gradually reduced to baseline (Figure 1(a)). CEPN was then removed and the high K^+^-induced contraction will restore to 26.7 ± 6.2% (*n* = 6) after 30 min. The results from 7 experiments yielded a half-maximal inhibition (IC_50_) of 35.4 ± 1.4 *µ*g/mL and a maximal relaxation of 103.4 ± 1.3% (Figure 1(b)). It is well known that high K^+^ induces depolarization resulting in the activation of VDCCs, which allows a Ca^2+^ influx to trigger contraction [27, 28]. This pathway was confirmed using a selective blocker of VDCCs, nifedipine (10 *µ*M), which completely blocked 80 mM K^+^-induced contractions (Figure 1(c)). CEPN inhibited precontraction, but it did not alter resting tension in 4 tracheal rings (Figure 1(d)). These data indicate that CEPN relaxed high K^+^-precontracted ASM via VDCC inhibition.

**Figure 2:**
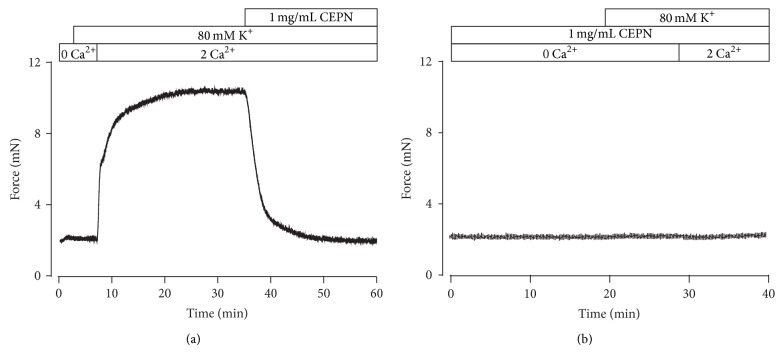
CEPN blocks high K^+^-evoked Ca^2+^ influx. (a) A representative tracing of 4 experiments. Under Ca^2+^-free conditions (0 Ca^2+^ and 0.5 mM EGTA), high K^+^ did not evoke contraction in a tracheal ring. After the restoration of 2 mM Ca^2+^, a sustained contraction occurred, which was fully inhibited by CEPN. (b) In the presence of 1 mg/mL CEPN, the identical experiments as above were performed. A Ca^2+^ restoration-provoked contraction was not noted. This experiment was conducted in 6 tracheal rings. These results suggest that CEPN inhibits Ca^2+^ influx.

The above results suggest that the relaxation induced by CEPN might be due to the termination of VDCC-mediated Ca^2+^ influx. This hypothesis was examined in the following experiments.  Figure 2(a) shows that high K^+^ did not induce contraction under Ca^2+^-free conditions (0 mM Ca^2+^ and 0.5 mM EGTA); however, contraction immediately occurred following Ca^2+^ restoration (2 mM), and CEPN (1 mg/mL) completely inhibited contraction. However, a Ca^2+^ restoration-induced contraction was not observed in the presence of 1 mg/mL CEPN (Figure 2(b)). This result suggests that CEPN-evoked relaxation of high K^+^-induced precontraction was completely dependent on the inhibition on VDCC-mediated Ca^2+^ influx.

### 3.2. CEPN Blocks VDCC Currents

**Figure 3:**
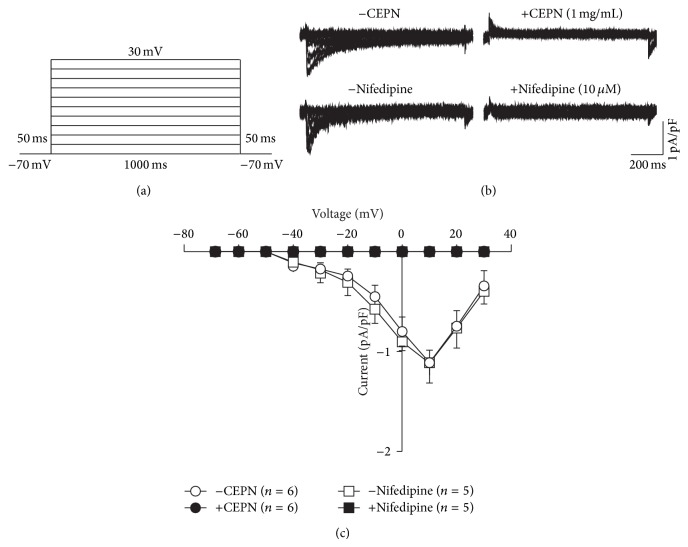
CEPN blocks VDCC currents. (a) The protocol used to measure VDCC currents in single ASMCs. (b) VDCC currents, recorded following depolarizations, were blocked by CEPN and nifedipine, respectively. (c) *I*-*V* relationships constructed based on the results of 5 to 6 experiments. These data indicate that CEPN blocks VDCCs.

We used patch-clamp techniques to measure VDCC currents (Ba^2+^ as the carrier charge) [25] with voltage steps from −70 to +30 mV to further confirm the ability of CEPN to block VDCCs (Figures 3(a) and 3(b)). Currents were abrogated following applications of CEPN and nifedipine. Current-voltage (*I*-*V*) curves were constructed based on the results of 5 to 6 experiments (Figure 3(c)). These data indicate that CEPN blocked VDCCs.

### 3.3. CEPN Inhibits ACh-Induced Precontraction

**Figure 4:**
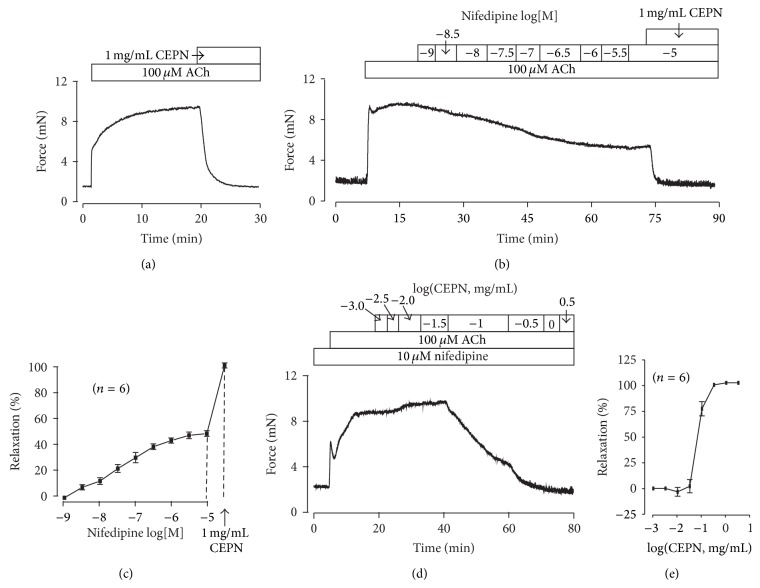
CEPN inhibits ACh-induced precontraction. (a) ACh induced a contraction in a tracheal ring that was inhibited completely by CEPN. This experiment was repeated in 6 rings. (b) Nifedipine partially reduced the ACh-induced contraction in a dose-dependent manner. The resistant contraction was inhibited by CEPN. The summary results from 6 experiments are shown in (c). (d) In the presence of nifedipine, ACh induced a typical contraction, which was dose-dependently inhibited by CEPN. The summary results from 6 experiments exhibited in (e). These results show that CEPN inhibits VDCCs and another pathway to induce relaxation.

We next observed whether CEPN inhibited ACh-induced precontraction.  Figure 4(a) shows that 1 mg/mL CEPN fully relaxed 100 *µ*M ACh-induced contraction (100.7 ± 0.4%, *n* = 6). If CEPN was removed, the ACh-induced contraction will recover to 92.2 ± 3.4% (*n* = 7) within 30 min. Moreover, the precontraction was partially blocked following additions of nifedipine and the resistant component was totally inhibited by 1 mg/mL CEPN (Figure 4(b)). The maximal inhibition by nifedipine and CEPN was 52.1 ± 2.2%and 105.2 ± 2.0%, respectively (*n* = 6; Figures 4(b) and 4(c)). These results indicate that VDCCs and an unknown pathway mediate CEPN-induced relaxation of ACh-induced precontraction. Our data have showed that CEPN blocked VDCCs to induce relaxation of high K^+^-evoked precontraction. Therefore, we only focused on defining the unknown pathway. We first observed the role of the unknown pathway in the dose-response of CEPN relaxation.  Figure 4(d) shows that VDCCs were blocked with 10 *µ*M nifedipine, and ACh was added to induce a steady-state contraction. Contractions continuously declined to resting levels following cumulative additions of CEPN. The dose-response curve was constructed based on 6 experiments (Figure 4(e)). These data demonstrate that CEPN completely reduced ACh-induced precontraction through the inhibition of VDCCs and an unknown pathway.

**Figure 5:**
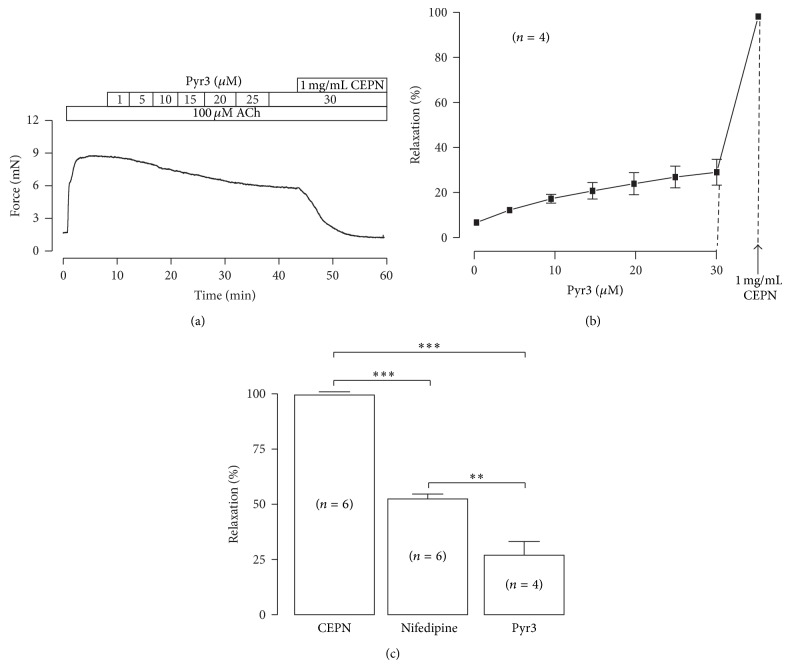
Pyr3 inhibits ACh-induced contraction. (a) Pyr3, a blocker of TRPC3 and Orai1 channels, partially inhibited ACh-induced contraction in a dose-dependent manner. The remained contraction was completely blocked by 1 mg/mL CEPN. The dose-response from 4 experiments is shown in (b). (c) Comparison of the relaxant effects of CEPN, nifedipine, and Pyr3 on ACh-induced contraction. ^**^ *P* < 0.01, ^***^ *P* < 0.001.

In addition, NSCCs predominantly mediate ASM contraction [29]. We used Pyr3 (an inhibitor of nonselective cation channel TRPC3 and Orai1 [30]) to examine whether TRPC3/Orai1 channels mediated ACh-induced contraction [31, 32] and found which was partially prevented by Pyr3 in a dose-dependent manner (Figures 5(a) and 5(b)). The Pyr3-induced maximal inhibition was 26.8 ± 6.2% (Figure 5(c),  *n* = 4, 30 *µ*M Pyr3) and the remained contraction was completely blocked by CEPN (Figures 5(a) and 5(b)). The Pyr3-induced relaxation was about half of that induced by nifedipine (52.1 ± 2.2%) as shown in Figure 5(c), suggesting that both VDCCs and NSCCs are involved in CEPN-induced relaxation.

### 3.4. CEPN Blocks ACh-Activated Ca^2+^ Influx

**Figure 6:**
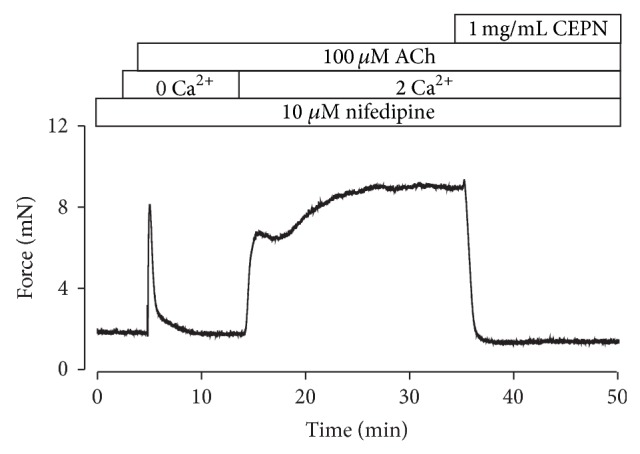
CEPN blocks ACh-evoked Ca^2+^ influx. Representative result of 4 experiments in the presence of nifedipine. Under Ca^2+^-free conditions (0 Ca^2+^ + 0.5 mM EGTA), ACh induced a fast transient contraction. Following the addition of 2 mM Ca^2+^, a strong sustained contraction occurred, which was entirely reversed by CEPN. These experiments indicate that CEPN inhibits the nifedipine-resistant Ca^2+^ influx activated by ACh.

Contraction is primarily dependent on the intracellular Ca^2+^ increase [33, 34]. Therefore, we investigated whether the unknown pathway involved Ca^2+^ influx. ACh induced a transient contraction in the presence of nifedipine (10 *µ*M) and Ca^2+^-free conditions (0 mM Ca^2+^ and 0.5 mM EGTA) (Figure 6). Addition of 2 mM Ca^2+^ triggered a sustained contraction, which was blocked by 1 mg/mL CEPN. These results indicate that the steady-state contraction induced by ACh was due to a nifedipine-resistant Ca^2+^ influx that was inhibited by CEPN. Therefore, the nifedipine-resistant Ca^2+^ influx was defined as the unknown pathway.

### 3.5. CEPN Inhibits NSCCs

**Figure 7:**
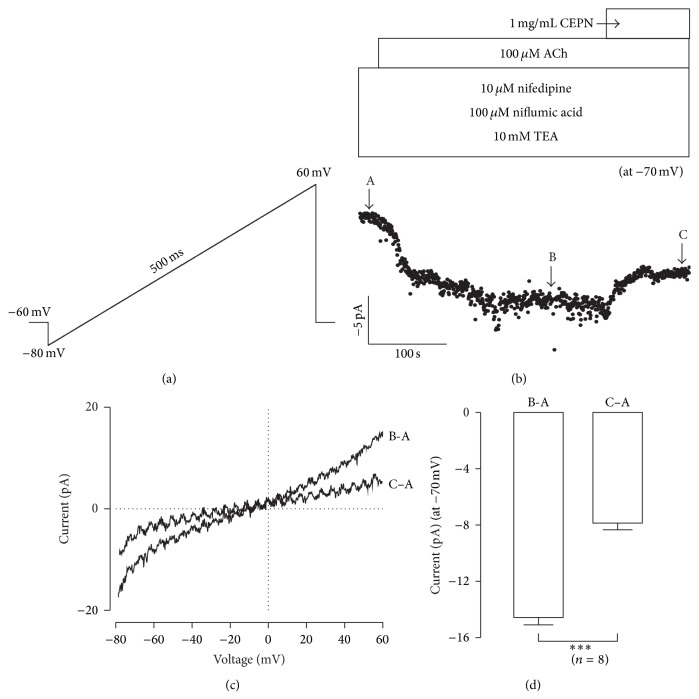
CEPN inhibits NSCC currents. (a) The ramp employed to record NSCC currents in single ASMCs. (b) Ramp current values at −70 mV were used to construct current-time traces, which exhibit ACh-induced NSCC currents that were partially blocked by CEPN. (c) The net ramp currents at time points B and C (shown in (b)). (d) At −70 mV, the mean currents at time points B and C. ^***^ *P* < 0.001. These data suggest that CEPN partially inhibits NSCCs.

We measured ACh-activated NSCC currents and observed the effects of CEPN on these currents to further determine the nature of the Ca^2+^ influx because ACh activates NSCCs to increase intracellular Ca^2+^ [25, 35, 36]. ACh-induced NSCC currents were purely isolated in the presence of nifedipine, NA and TEA, and were recorded by a ramp (Figure 7(a)). NSCC currents were partially blocked by CEPN (Figure 7(b)). Two representative ramp current traces at time points b and c (indicated in Figure 7(b)) are shown in Figure 7(c). The leak ramp currents at time point a (indicated in Figure 7(b)) were subtracted. The mean values of current amplitudes at −70 mV were −14.5 ± 0.5 pA and −7.8 ± 0.5 pA at time points b and c, respectively (*n* = 8, Figure 7(d)). These data indicate that CEPN partially inhibited ACh-induced NSCCs.

## 4. Discussion

The present study demonstrated that CEPN induced strong relaxation in precontracted mouse ASM induced by high K^+^ and ACh through blockade of Ca^2+^ influx mediated by VDCCs and NSCCs. This finding suggests that CEPN may be a potential bronchodilator.

High K^+^ induces membrane depolarization, which opens VDCCs that mediate extracellular Ca^2+^ influx and induce contractions [27, 28]. The present study showed that CEPN completely inhibited high K^+^-induced contractions in ASM (Figures 1(a) and 1(b)), which suggests that this inhibition was due to the blockade of VDCCs by CEPN. We designed three different experiments to further support this result and showed that the selective blocker of VDCCs nifedipine totally inhibited high K^+^-evoked contractions (Figure 1(c)), CEPN inhibited Ca^2+^ influx-induced contractions (Figure 2), and CEPN directly blocked VDCC-mediated currents (Figures 3(b) and 3(c)). These data demonstrate that CEPN blocked VDCCs, which terminated the Ca^2+^ influx that leads to the relaxation of high K^+^-induced precontracted mouse ASM.

The muscarinic receptor agonist ACh activates both VDCCs and NSCCs, which leads to Ca^2+^ influx and an increase in intracellular Ca^2+^ to trigger ASM contraction [35, 36]. This pathway was demonstrated in our recent results [25]. The present findings implied that CEPN-induced relaxation of ACh-evoked contraction (Figure 4(a)) resulted from the inhibition of both VDCCs and NSCCs by CEPN. This is because the fact that (1) CEPN completely blocked ACh-induced contraction (Figure 4(a)), (2) the selective blocker of VDCCs, nifedipine, partially inhibited ACh-induced contraction and the remaining component was blocked by CEPN (Figures 4(b), 4(c), 4(d), and 4(e)), and the latter was due to the inhibition of Ca^2+^ influx by CEPN (Figure 6), (3) ACh-induced NSCC currents, which mediate Ca^2+^ influx, were partially blocked by CEPN (Figure 7), and (4) ACh-induced contraction was partially blocked by Pyr3, a selective blocker of nonselective cation channel TRPC3 and Orai (Figure 5). Therefore, we conclude that CEPN blocks VDCC- and NSCC-mediated Ca^2+^ influx to result in relaxation of ACh-precontracted ASM.

## 5. Conclusions

CEPN induced relaxation of precontracted mouse ASM through the inactivation of VDCCs and NSCCs. This study supports the development of new drugs from CEPN to treat airway hyperresponsiveness in asthmatic and COPD patients. Further investigation will be required to identify the components that are responsible for the relaxation action.
